# Preparation and characterization of an amphiphilic polyamide nanofiltration membrane with improved antifouling properties by two-step surface modification method

**DOI:** 10.1039/c8ra00637g

**Published:** 2018-04-10

**Authors:** Huimin Ruan, Bin Li, Jianbing Ji, Arcadio Sotto, Bart Van der Bruggen, Jiangnan Shen, Congjie Gao

**Affiliations:** Center for Membrane Separation and Water Science & Technology, Ocean College, Zhejiang University of Technology Hangzhou 310014 PR China shenjn@zjut.edu.cn; College of Chemical Engineering, Zhejiang University of Technology Hangzhou 310014 PR China; Rey Juan Carlos University 28942 Fuenlabrada-Madrid Spain; Department of Chemical Engineering, KU Leuven Celestijnenlaan 200F B-3001 Leuven Belgium

## Abstract

Membrane fouling is an urgent problem needing to be solved for practical application of nanofiltration membranes. In this study, an amphiphilic nanofiltration membrane with hydrophilic domains as well as low surface energy domains was developed, to integrate a fouling-resistant defense mechanism and a fouling-release defense mechanism. A simple and effective two-step surface modification of a polyamide NF membrane was applied. Firstly, triethanolamine (TEOA) with abundant hydrophilic functional groups was grafted to the membrane surface *via* reacting with the residual acyl chloride group of the nanofiltration membrane, making the nanofiltration membranes more hydrophilic; secondly, the 1*H*,1*H*,2*H*,2*H*-perfluorodecyltrichlorosilane (PFTS), well-known as a low surface energy material, was covalently grafted on the hydroxyl functional groups through hydrogen bonding. Filtration experiments with model foulants (bovine serum albumin (BSA) protein solution, humic acid solution (HA) and sodium alginate solution (SA)) were performed to estimate the antifouling properties of the newly developed nanofiltration membranes. As a result of surface modification proposed in this study the antifouling properties of an amphiphilic modified F-PA/PSF membrane were enhanced more than 10% compared to the PA/PSF specimen in terms of flux recovery ratio.

## Abstract 2

Schematic diagram of amphiphilic NF membrane by a two-step modification.

## Graphical abstract

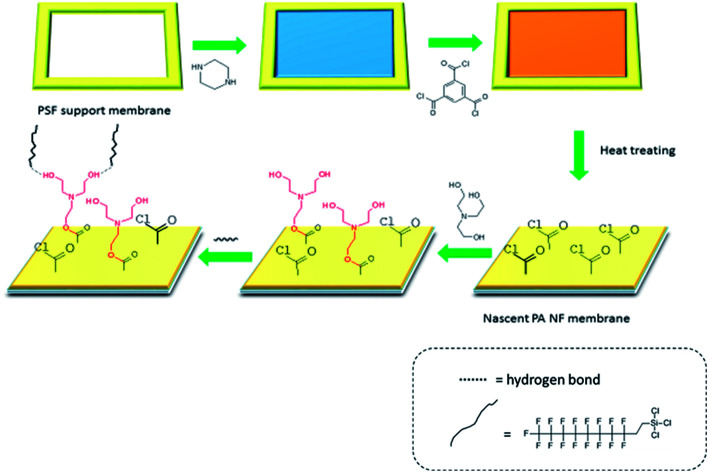

## 1. Introduction

Resource depletion and environmental pollution have become an exigent global issue that threatens daily life. Membrane technology becomes attractive in this context, as it is an energy-saving and environmentally friendly technology. Nanofiltration membranes (NF), which have a high permeation flux under low operational pressures, have been applied in water purification, pretreatment for seawater desalination, removal of heavy metals, textile dyeing and other fields.^1–3^ Thin-film composite (TFC) membranes are the most popular commercial NF membranes which usually consist of an ultra-thin selective layer upon the surface of a porous flat sheet substrate membrane through the interfacial polymerization (IP) process, using diamine and acyl chlorides.^4,5^ However, membrane fouling is a bottleneck for the practical application of NF in water and wastewater treatment. Fouling usually causes a significant loss of productivity and increased operational cost.^6^ Foulants in the water (like colloid substances, bacteria, and nature organic materials) can unsuitably absorb and deposit onto the membrane surface or into the pores and pore walls and thus cause membrane fouling,^7,8^ which results in a flux decline during filtration operation and a shorter lifetime. Many researchers^9–11^ have suggested that the dominant reason of membrane fouling is the intrinsic hydrophobicity of the membrane materials. Besides, the roughness and charge of the surface, feed solution chemistry and process conditions also have an important influence on the membrane fouling.^8^

The pores of NF membranes are very small (∼1 nm), so that fouling is mainly manifested as surface fouling.^12^ Currently, several surface modification methods like surface grafting,^13^ surface blending^14^ and surface coating^15^ have been used to improve the antifouling performance of NF membranes. Commonly, surface covalent grafting of NF membrane is performed by using residual acyl chloride on the active layer to react with other functional groups capable of reacting with them. Zhu *et al.*^16^ studied the structure and properties of TFC hollow fiber NF membranes grafted with poly (amidoamine) dendrimer (PAMAM). The hydrophilicity and water permeation flux increased with the PAMAM grafting, while the salt rejection was hardly affected. The effect of the PAMAM grafted TFC membrane on the removal of heavy metal ions like Pb^2+^, Cu^2+^, Ni^2+^, Cd^2+^, Zn^2+^ and As^5+^ was also significant, the rejection was over 99%.

At present, antifouling membranes can be divided into several categories according to different criteria, on the basis of hydropathy property of membranes surfaces, the membranes can be classified into three categories:^17^ hydrophilic antifouling surfaces, hydrophobic self-cleaning surfaces and amphiphilic surfaces. Hydrophilic modification of NF membranes is one of the main ways to enhance the antifouling properties of a membrane. It is based on the fact that the organic matters in the water, which are hydrophobic in nature, are more likely to attach on a membrane surface because of hydrophobic interactions. The advanced hydrophilicity of membrane surface is beneficial to mitigate these molecular interactions.^18^ Furthermore, the hydrophilic membrane surface promotes the formation of a hydration layer, which restricts the pollutant adsorption or deposition on the membrane surface.^19^ Many hydrophilic materials have been used to prepare highly antifouling NF membranes, for example, poly(ethylene glycol) (PEG)-based materials,^20,21^ zwitterionic materials;^22,23^ among the most popular inorganic nanoparticles are titanium dioxide (TiO_2_),^8,24^ graphene oxide (GO),^25,26^ and multi-wall carbon nanotubes (MWCNTs).^14,27,28^ The strategy of hydrophilic modification represents a fouling-resistance defense mechanism.^29–31^ Unlike hydrophilic surfaces, hydrophobic membrane surfaces follow another mechanism: the fouling-release defense mechanism.^32–34^ This works through the development of a low surface energy layer barrier on the membrane surface using silicone-based^35,36^ and fluorine-based materials,^37,38^ with a surface energy of about 22 mJ m^−2^ and 10–18 mJ m^−2^, respectively. Since the low surface energy characteristics of self-cleaning surfaces can make the intermolecular forces between pollutants and surfaces minimization, so that the pollutants are easily removed through low hydrodynamic shear force or simple mechanical cleaning, yielding an attractive “fouling-release” property.^39^

Although many attempts have been made to enhance the antifouling abilities of membrane surfaces, there are still significant problems to be addressed. With respect to hydrophilic modified membranes, the hydrophilic segments (like PEG) would be easily washed out of the matrix under the large drag force from the filtration pressure, because of their linear structures and low compatibility with the membrane matrix.^40,41^ The surface of the membrane is still exposed to the risk of pollutant adsorption and deposition, thus reducing the flux. Therefore, the hydrophilic modification of NF membrane surfaces may is not a foolproof solution to solve the fouling effects. As for the hydrophobic modified film, one of the biggest problems is that the introduction of low surface energy materials may cause the water flux decline. Thus, the amphiphilic surfaces containing both hydrophilic and low surface energy domains would be good candidates for membrane modification. The presence of hydrophilic domains guaranties high water fluxes and low flux decline as result of hydraulic layer formation that mitigates the interaction between potential pollutants and the membrane surface. On the other hand, the hydrophobic anchoring groups are entangled with the hydrophobic membrane matrix because of the well compatibility, correlative hydrophilic segments can be stabilized in the membrane surface, thus weakens the interaction force between pollutants and membrane surface.^40,42^ The amphiphilic modification of NF membrane may thus meet the antifouling requirements of a much broader variety of pollutants.

For the past few years, many researchers have paid attention to develop the amphiphilic surfaces on ultrafiltration membranes with multi-defense fouling mechanisms.^43,44^ The common method is to fabricate an amphiphilic polymer and then blend it into the casting solution to prepare antifouling membranes by different methods, such as surface segregation,^31,36^ phase separation,^41,45^ dip-coating^46^ or phase inversion.^40^ However, due to the large size of the synthesized copolymer molecules and much smaller pore size of the NF membrane, the abovementioned methods are not suitable for the preparation of the amphiphilic antifouling NF membrane taking into account the possible blockage of membrane pores. Thus, a new way is required to prepare amphiphilic NF membrane surfaces. Zhang *et al.*^17^ fabricated an amphiphilic NF membrane by a two-step surface modification of a polyamide NF membrane, this amphiphilic NF membrane contains both hydrophilic domains and low surface energy domains, which can combine the fouling-resistance defense mechanism and the fouling-release defense mechanism. Triethylenetetramine with primary amine groups was firstly introduced to the membrane surface by reacting with unreacted acyl chloride on the surface of polyamide NF membrane. And then 2,2,3,4,4,4-hexafluorobutylmethacrylate was covalently grafted to the surface of the membrane by a Michael addition reaction with these primary amine groups. The antifouling experiments of BSA solution, HA solution and SA solution turned out that the flux recovery rate of the amphiphilic membrane was higher than of polyamide NF membrane, therefore possessed better antifouling property. However, this procedure is performed under harsh reaction conditions, which needs be carried out at a higher temperature, and the reaction time is relatively long. This will undoubtedly increase the energy consumption, and limit its industrial application. In this study, a simple and effective method is proposed avoiding the drawbacks mentioned above. The process involved in this approach can be performed at room temperature with the enhanced reaction kinetics.

An amphiphilic nanofiltration membrane with hydrophilic domains and low surface energy domains was developed through a two-step surface modification. First, the polyamide NF membrane was fabricated by interfacial polymerization of piperazine (PIP) and trimesoyl chloride (TMC). Then TEOA having abundant hydrophilic functional groups, is cost-effective, environment-friendly and easily-obtained. It can be grafted to the surface by reacting with the residual acyl chloride group of NF membrane, which makes the NF membranes more hydrophilic. Finally, the PFTS which is usually known as a low surface energy material was covalently grafted on hydroxyl functional groups through hydrogen bonding. Morphology of the prepared membranes were characterized using atomic force microscopy (AFM), scanning electron microscopy (SEM), *etc.* Separation and rejection properties were investigated by filtration experiments of salts and PEGs. Filtration experiments with model foulants (BSA protein solution, HA solution and SA solution) were performed to estimate the antifouling property of the prepared NF membranes.

## 2. Materials and methods

### 2.1 Materials and chemicals

A flat-sheet nonwoven-reinforced polysulfone (PSF) support membrane with average molecular weight cut-off (MWCO) of 80 000 g mol^−1^ was supplied by Hangzhou Water Treatment Technology Development Center (Hangzhou, China). Piperazine (PIP, 99%), *n*-hexane (≥97%), trimesoyl chloride (TMC), sodium dodecyl sulfate (SDS, analytically pure), triethanolamine (TEOA, 98%), bovine serum albumin (BSA, analytically pure), sodium alginate (SA, analytically pure), humic acid (HA, analytically pure), 1*H*,1*H*,2*H*,2*H*-perfluorodecyltrichlorosilane (PFTS, 95%) and polyethylene glycol (PEG) with different molecular weights (*M*_w_ = 200, 300, 400, 600, 800 and 1000 Da) were purchased from Macklin (Shanghai, China). Alcohol, sodium sulfate (Na_2_SO_4_), and sodium chloride (NaCl) were purchased from Shanghai Lingfeng Chemical Reagent Co. Ltd, China. All reagents were used as received without further purification. Pure water used throughout in this work was derived from a Milli-Q system (Millipore, US).

### 2.2 Membrane preparation

**Fig. 1:**
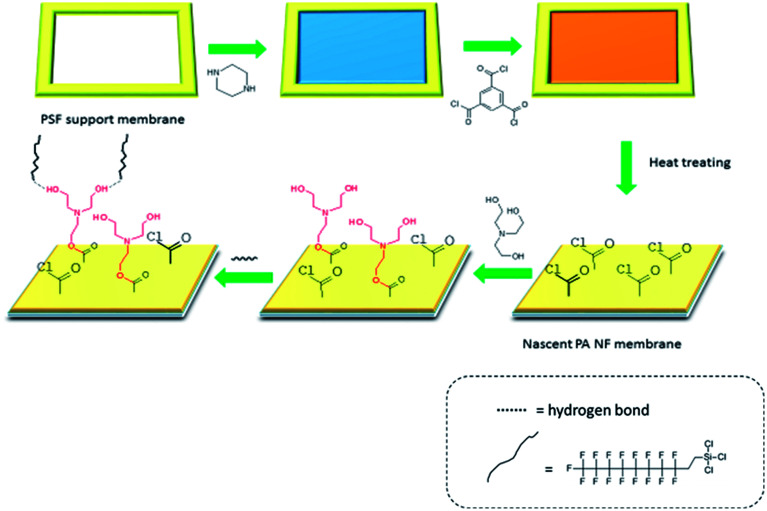
Schematic diagram of amphiphilic NF membrane by a two-step modification.

Fig. 1 shows the preparation procedure of the amphiphilic NF membrane, which contained a two-step surface modification of NF membrane. The nascent polyamide NF membrane was fabricated by interfacial polymerization of PIP and TMC on the PSF support membrane. The PSF support membrane which fixed by polyfluortetraethylene framework was firstly soaked into 0.5 wt% PIP aqueous solution containing 0.1 wt% SDS for 2 min, and then the aqueous solution was poured out. The membrane surface was rinsed with pure water to remove the remaining liquid droplets, followed by air dried at room temperature. Subsequently, the PIP impregnated membrane surface was brimming with 0.1 wt% TMC organic solution (*n*-hexane) for 1 min, and then the organic solution was poured out. The membrane surface was rinsed thoroughly with *n*-hexane, heat treatment of 60 °C for 10 min was further carried out to make sure completion of the polymerization reaction. The resultant NF membrane was named PA/PSF membrane. On this basis, the amphiphilic membrane was prepared by a two-step modification.

Firstly, the surface of a newly prepared PA/PSF membrane was coated with TEOA ethanol solution for 2 min and washed thoroughly with ethanol to remove the remaining TEOA solution and then pure water to remove the residual ethanol. Membranes prepared with TEOA contents of 1, 2, 3, 4 and 5% (v__TEOA__/v_solution_) are referred to as H_1_-PA/PSF, H_2_-PA/PSF, H_3_-PA/PSF, H_4_-PA/PSF and H_5_-PA/PSF, respectively. The resultant hydrophilic modified membranes were stored in pure water. Secondly, the surface of the hydrophilic modified membrane was immersed in PFTS alcohol solution with for 2 min. The membrane surface was flashed with alcohol to remove the remaining PFTS solution and then pure water to remove the remaining ethanol, followed by drying at 80 °C for 10 min. After heat treatment, the fabricated membrane (designated as the F-PA/PSF membrane) was stored in pure water for the next experiment.

### 2.3 Membrane characterization

The NF membrane was submerged in water for 24 hours prior to the determination of chemical composition analysis and morphologies. Attenuated Total Reflectance-Fourier transform Infrared spectroscopy (ATR-FTIR, Nicolet 6700) and X-ray photoelectron spectroscopy (XPS, Kratos AXIS Ultra DLD, Japan) were used to characterize the chemical composition and structure of membrane surface before and after modification. The samples were scanned from 400 to 4000 cm^−1^ by ATR-FTIR with a resolution of 1 cm^−1^ for each spectrum. XPS were operated at constant pass energy of 100 eV for wide scans and 20 eV for detailed scans setting the O1s peak at BE 528.8 eV. The morphological characteristics of the membranes were observed with a scanning electron microscope (SEM, HITACHI, S4700A). The membrane samples were dried under vacuum and sputter-coated with gold for producing electric conductivity before observation. Atomic force microscopy (AFM, Bruker Dimension station) was used to characterize the surface topology of the membranes with a scanning area of 10 μm × 10 μm.

The water contact angle of pristine and modified membrane surfaces was measured using a contact angle goniometer (OCA-20, Data-physics, Germany). For each membrane sample, at least five measurements were taken at different locations at room temperature and the average value was reported. The surface charge of the membranes were analyzed by an electrokinetic analyzer (SurPASS 3, Anton Paar, Austria) with 1 mM KCl as the electrolyte solution at a pH of 6.5–7.5 at room temperature. Zeta potential of the membrane was measured by an electrokinetic analyzer (SurPASS 3, Anton Paar, Austria) with 1 mM KCl as the electrolyte solution at a pH of 3.1–9.7 at room temperature.

### 2.4. Membrane filtration testing

The permeability and rejection tests were measured in a batch cross-flow NF membrane test unit at 25 °C. All the membrane samples with effective area 19.6 cm^2^ (*A*) were pre-compacted at 1.1 MPa with pure water for 90 min to obtain a stable flux before testing and then evaluated for 30 min at a pressure of 1.0 MPa. The penetrating fluid is collected for the calculation of the water flux (*J*) and salt rejection (*R*) as follows:1
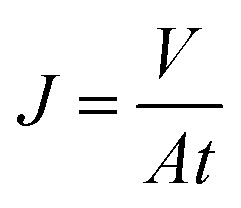
2
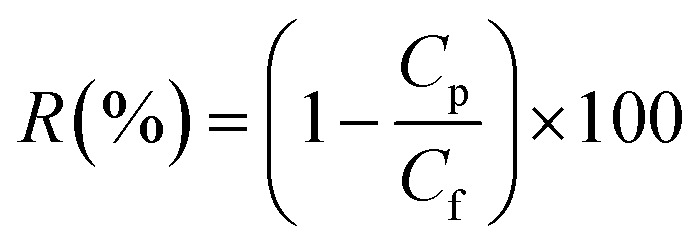
where *t* is the permeation time (h). *C*_p_ and *C*_f_ are the solute concentrations in permeate and feed, respectively. 1.0 g L^−1^ of Na_2_SO_4_ or NaCl solution was used to estimate the rejection performance of membrane. Molecular weight cut-off of membrane was tested using 1 g L^−1^ PEGs aqueous solution with different molecular weights (200, 300, 400, 600, 800, and 1000 Da). A total organic carbon analyzer (TOC Analyzer, Shimadzu TOC-VCNP) were used to determine the concentrations of the PEGs. The salt concentrations were measured by a conductivity meter (DDSJ-308A, Leizi, China), since the electric conductivity is proportional to the total salt concentration in a certain range. The average value of the three sets of parallel experimental data was used to calculate water flux and salt rejection.

### 2.5 Antifouling performance of NF membranes

Experiments with model foulants (BSA protein solution, HA and SA solution) were performed to estimate the antifouling property of NF membranes, which were chosen to represent protein, natural organic matter (NOM) and polysaccharide, respectively. The concentration of foulants solution was 1 g L^−1^. Before the antifouling experiment, the pure water flux (*J*_W1_) of NF membranes was measured at 1.0 MPa, and then the solution reservoir was filled with 1.0 g L^−1^ of foulant solution. Fouling tests were performed under the same pressure, the penetrating fluid was collected every 30 min for 24 h, a steady flux (*J*_p_) for foulant was obtained over a period of time. After that, the permeation returned to the feed tank to maintain a constant feed concentration. When the filtration of the foulants solution is finished, the membrane was cleaned with pure water for 30 minutes and the recovery flux after washing was measured (*J*_W2_). The flux recovery ratio (FRR) and total flux decline ratio (DR_t_) were used to signify the antifouling effect of NF membranes, defined as follows:^47^3
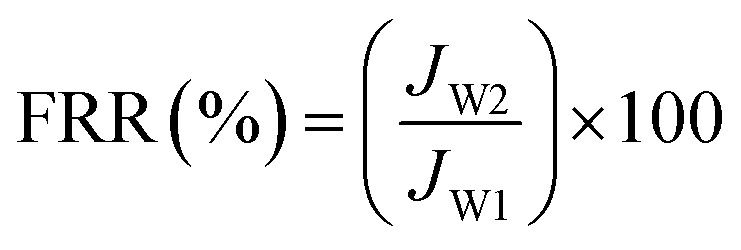
4
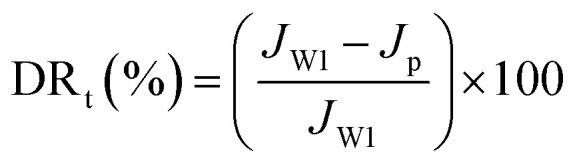


## 3. Results and discussion

### 3.1 Characterization of NF membrane

**Fig. 2:**
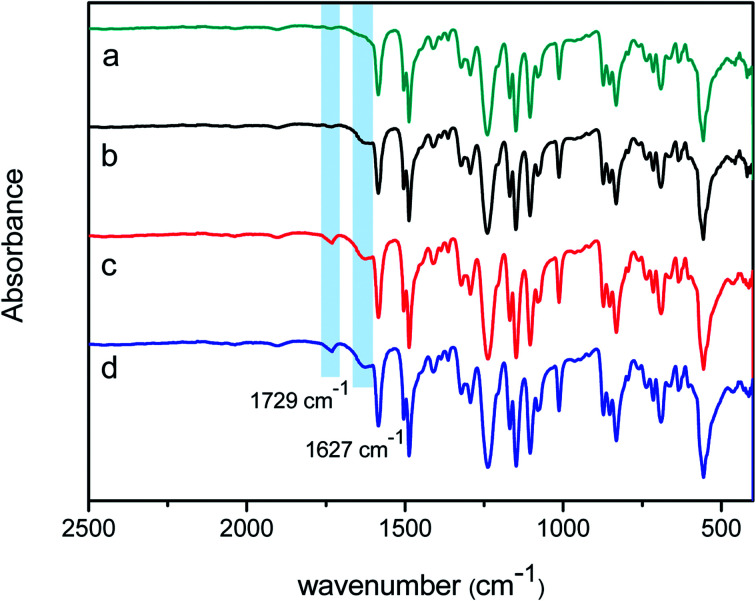
ATR-FTIR spectra of the surfaces of (a) PSF membrane; (b) polyamide NF membrane PA/PSF; (c) TEOA-grafted NF membrane H_4_-PA/PSF and (d) PFTS-grafted NF membrane F-PA/PSF.

ATR-FTIR and XPS were used to understand the chemical structure of the skin layer of the NF membranes. Fig. 2 presents the ATR-FTIR spectra of the surfaces of PSF support membrane (a), nascent NF membrane PA/PSF (b), TEOA-grafted NF membrane H_4_-PA/PSF (c) and PFTS-grafted NF membrane F-PA/PSF (d). Compared with the spectrum of the PSF support membrane, an apparent absorption peak at 1627 cm^−1^ corresponding to C=O stretching vibration of amide I appears in spectra (b), (c) and (d), confirming the formation of polyamide active layer after IP process. The new peak at 1729 cm^−1^ of membrane H_4_-PA/PSF (spectra (c)) is ascribed to *ν*_C=O_ of ester compound, which is formed by the esterification reaction between the hydroxyl groups of TEOA and the residual acyl chloride groups on the polyamide active layer. However, there is no difference between spectrum (c) and spectrum (d), this may be because the concentration of PFTS in membrane F-PA/PSF is too low to be detected by ATR-FTIR.

**Fig. 3:**
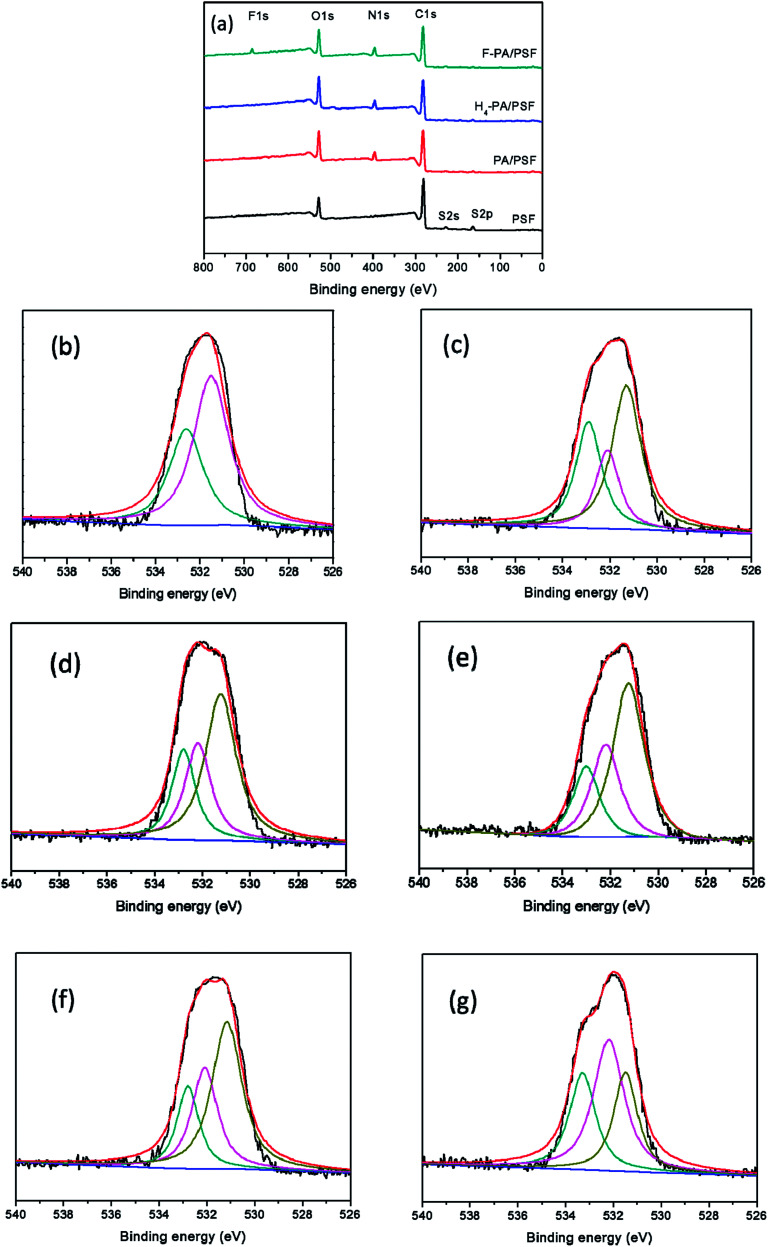
XPS scans of membranes PSF, PA/PSF, H_4_-PA/PSF and F-PA/PSF membrane (a) and (b) O1s core level XPS spectra of membranes PA/PSF; (c) H_1_-PA/PSF; (d) H_2_-PA/PSF; (e) H_3_-PA/PSF; (f) H_4_-PA/PSF and (g) H_5_-PA/PSF.

To make sure that the PFTS was successfully grafted onto the TEOA-modified membrane, an XPS characterization was further accomplished. Fig. 3 shows the XPS scans of PSF, PA/PSF, H_4_-PA/PSF and F-PA/PSF membrane. To distinguish the chemical changes of TEOA-modified NF membrane with different content of TEOA, O1s core-level spectra of membranes H_1_-PA/PSF, H_2_-PA/PSF, H_3_-PA/PSF, H_4_-PA/PSF and H_5_-PA/PSF are shown in (b), (c), (d), (e) and (f). Compared with the PSF membrane, the appearance of N1s signal of PA/PSF membrane in (a) confirmed the formation of a polyamide active layer on the PSF membrane. The O contents of H_4_-PA/PES membrane is higher than of PA/PSF membrane, while the N and C contents were lower (O: 19.47%→22.13%; N: 8.85%→7.73%; C: 71.68%→70.34%), as the grafted TEOA during first-step reaction. A new observed signal for F1s of F-PA/PSF membrane is an evidence that the PFTS has been successful grafted to the NF membrane surface. In the O1s core-level spectra of membranes, the peaks at approximately binding energy of 531.5, 532.2 and 533.3 eV are attributed to the N–C=O*/O–C=O*, C–O*–H and O=C–O* functional groups,^48^ respectively. The C–O*–H is introduced by TEOA, the conversion percentage (CP, %) of carboxyl groups into ester bonds is calculated to quantitatively represent the grafting ratio of TEOA. The calculation formula of CP is as follows:^49^

5 CP = (1/2*A*_C–O*–H_/*A*_O=C–O*_) × 100%

where *A* is the peak areas. The calculated CP of membranes H_1_-PA/PSF, H_2_-PA/PSF, H_3_-PA/PSF, H_4_-PA/PSF and H_5_-PA/PSF are 34.1%, 55.0%, 71.4%, 79.7% and 83.3%, respectively.

**Fig. 4:**
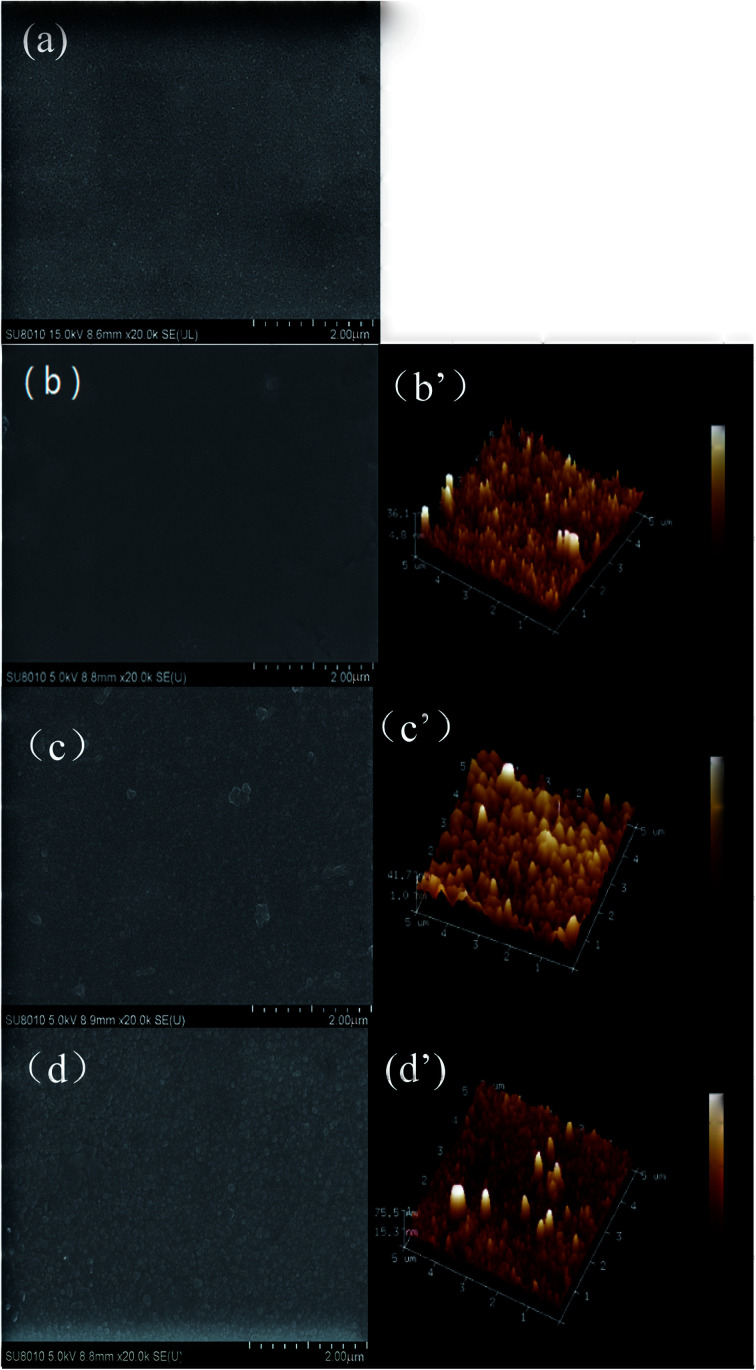
The surface morphology of (a) PSF; (b) PA/PSF; (c) H_4_-PA/PSF; and (d) F-PA/PSF membranes; three-dimensional AFM images of (b′) PA/PSF; (c′) H_4_-PA/PSF; and (d′) F-PA/PSF membranes.

The surface morphologies of the PSF and PA NF membranes were observed by SEM and AFM. As shown in Fig. 4, the PA/PSF membrane had a smooth surface (b) compared with the support PSF membrane (a). After the first-step of the modification, the H_4_-PA/PSF membrane surface appeared some small nodular structures (c), may be due to the aggregation of TEOA. After the second-step of the modification (d), the surface of the F-PA/PSF membrane was much rougher. AFM analysis was further carried out for quantitative investigation, and the results are shown in Fig. 4. It was found that the surface roughness of membranes slightly changed: the *R*_a_ values of membrane PA/PSF, H_4_-PA/PSF and F-PA/PSF were 7.7 ± 0.3, 10.4 ± 1.4, and 12.2 ± 3.1, respectively. A smooth surface restricts the adsorption and adhesion of pollutants, which is favorable to antifouling property. The results were in agreement with the SEM morphology observations.

**Fig. 5:**
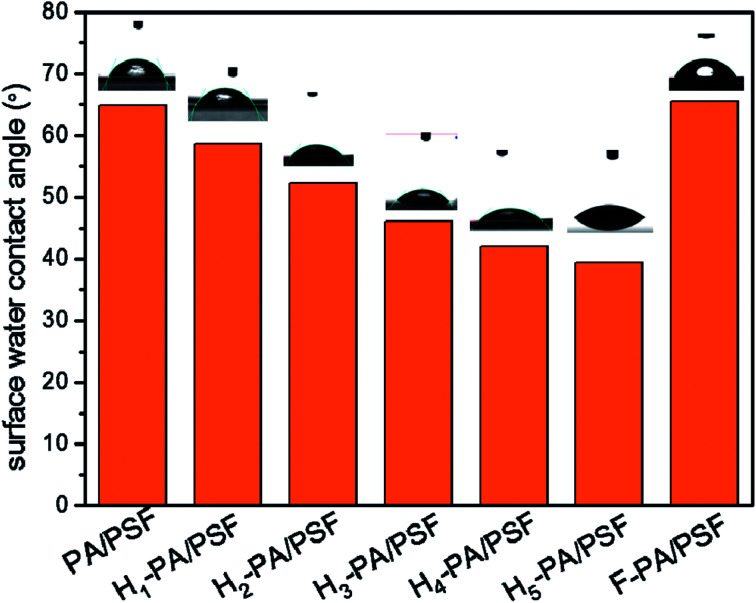
Water contact angles of NF membranes. (The error in contact angle measurement was <2°).

**Fig. 6:**
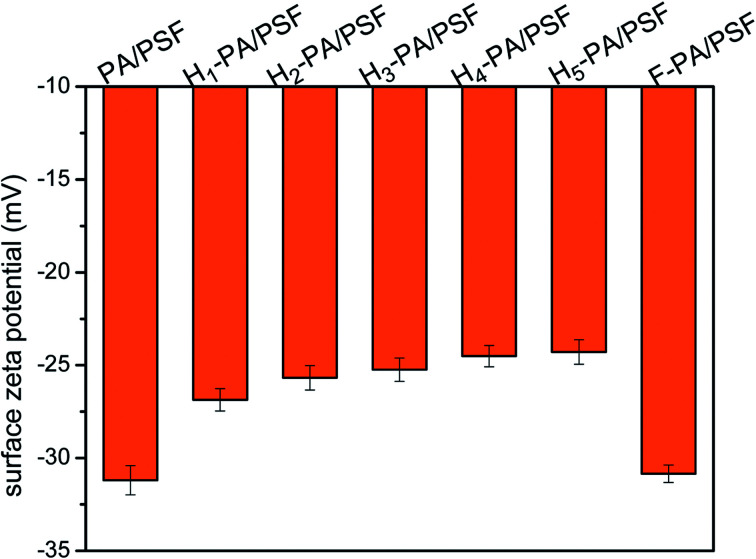
Zeta potentials measurements for PA NF membranes (pH = 7.0 ± 0.2).

As shown in Fig. 5, the hydrophilicity of the membrane surface was measured by the surface water contact angle (WCA). The value of WCA decreased after the TEOA grafting, which means that the hydrophilicity of the membrane surface was increased. The enhancement of membrane hydrophilicity is caused by the increase of hydroxyl groups density in TEOA. This effect is beneficial for the water permeation as well as to mitigate the hydrophobic pollutant adsorption onto the membrane surface. This is conducive to the improvement of permeation flux and the reduction of fouling by hydrophobic substances. The membrane surface charge was evaluated through the zeta potential measurements at neutral pH, depicted in Fig. 6. Clearly that all the surface were negatively charged. The zeta potential of TEOA-modified membrane was less negatively charged in comparison to PA/PSF membrane. As increasing the TEOA content, the zeta potential negative charge decreases. This effect can be understood taking into account that some acyl chloride groups reacted with the hydroxyl functional groups of TEOA, thus the amount of the acyl chloride groups for hydrolysis decreased as result of increased amount of TEOA.^48^ The zeta potential of the membranes decreased after a two-step modification, which could be attributed to the introduction of HFBM. The substantial changes in the zeta potential of the membranes indicated the success of the chemical modification.

### 3.2 Separation performance of membranes

**Fig. 7:**
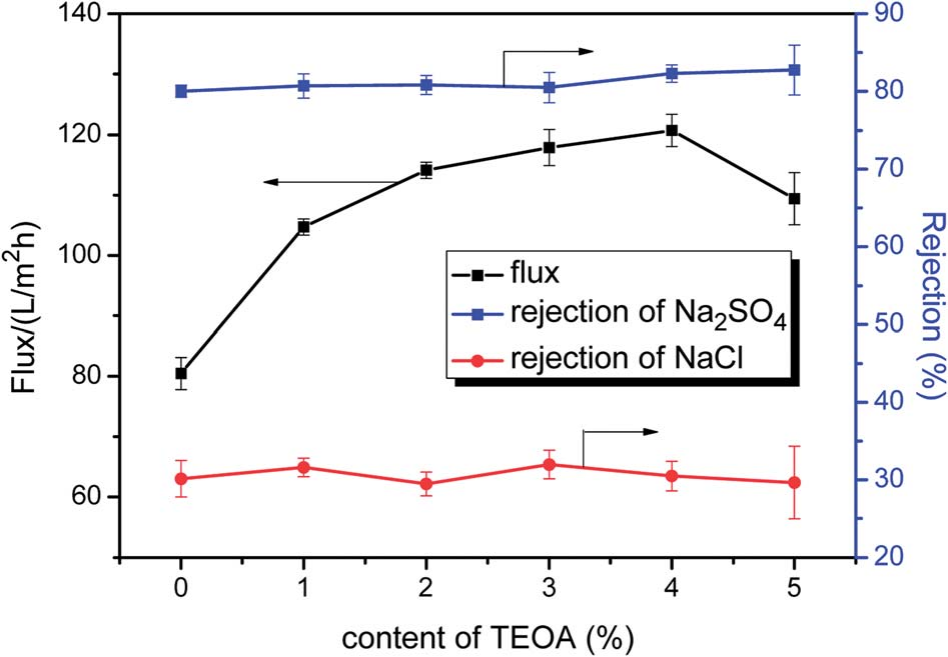
Effect of the TEOA content on permeation flux and salt rejection of PA/PSF membranes during first-step modification (reaction time fixed at 2 min, tested under 1.0 MPa and 25 °C).

**Fig. 8:**
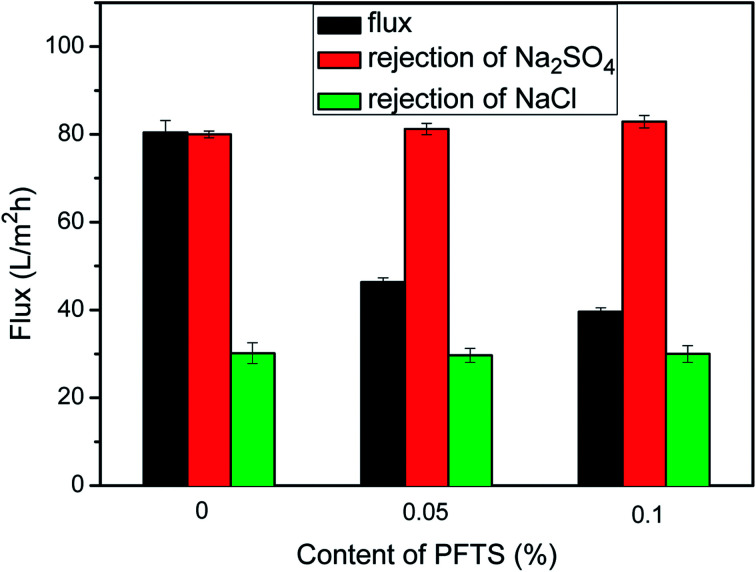
Effect of PFTS content on permeation flux and salt rejection of PA/PSF membranes during second-step modification (reaction time 2 min, reaction temperature 80 °C, testing was performed at 1.0 MPa and 25 °C).

The preparation conditions during two-step surface modification (such as concentration of TEOA and PFTS) were examined to deduce the optimal separation performance of the modified NF membrane. The effect of the TEOA concentration on the permeation flux as well as on the NaCl and Na_2_SO_4_ rejection of the PA/PSF membranes during the first-step modification is displayed in Fig. 7. The water flux increased visibly when the TEOA concentration increased from 0 to 4%, and then decreased when the TEOA concentration increased to 5%. On the other hand, the NaCl and Na_2_SO_4_ rejection changes only slightly. The improved surface hydrophilicity of the TEOA-modified membranes is the main reason to the improvement of water flux. In addition, ethanol as a solvent facilitates the improvement of flux. This is because the polyamide layer and the PSF support membrane have different swelling degree in the ethanol solvent (swelling degree of the polyamide layer is larger than the support membrane), so the gap between the two layers increases in the ethanol solvent, which is favorable to the passage of more water molecules.^17,50^ The slight fluctuation in salt rejection is due to the lower electrostatic repulsive effect caused by the reduced surface negative charge.^51^ The effect of the PFTS concentration on the permeation flux as well as on the NaCl and Na_2_SO_4_ rejection of the PA/PSF membranes during second-step modification is presented in Fig. 8. The flux clearly declined after PFTS was grafted due to the hydrophobicity of PFTS. When the content of PFTS increases from 0.05% to 1.0%, the flux decreases from 46.3 ± 1.0 L (m^2^ h)^−1^ to 39.6 ± 0.9 L (m^2^ h)^−1^, while the rejection of Na_2_SO_4_ and NaCl remains constants about 80% and 30%, respectively.

**Fig. 9:**
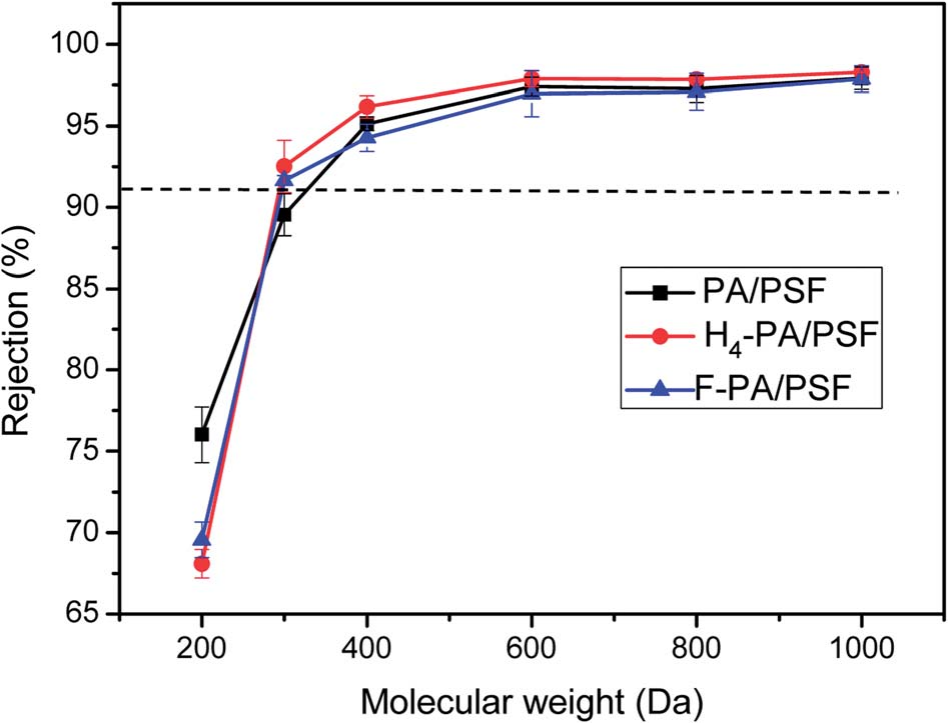
Rejections to PEGS of PA/PSF, H_4_-PA/PSF and F-PA/PSF membranes tested with 1 g L^−1^ aqueous solution at 1.0 MPa and 25.0 °C.

Molecular weight cut-off (MWCO) values of the membranes were also determined using PEGs as model solutes. Fig. 9 shows the rejection of PA/PES, H_4_-PA/PSF and F-PA/PSF membranes for six PEGs solutions with different molecular weights (200, 300, 400, 600, 800, and 1000 Da). All tests were performed at 1.0 MPa and 25 °C. According to the solute rejection curve, the MWCO of the PA/PSF, H_4_-PA/PSF and F-PA/PSF membranes were around 305 Da, 290 Da and 290 Da, respectively, indicating that the surface modification did not alter the pore size of membranes. The rejection performance of modified membrane is hardly affected by the chemical modification proposed in this study.^48,52^ This is consistent with the observed salt rejections.

### 3.3 Antifouling property of membranes

**Fig. 10:**
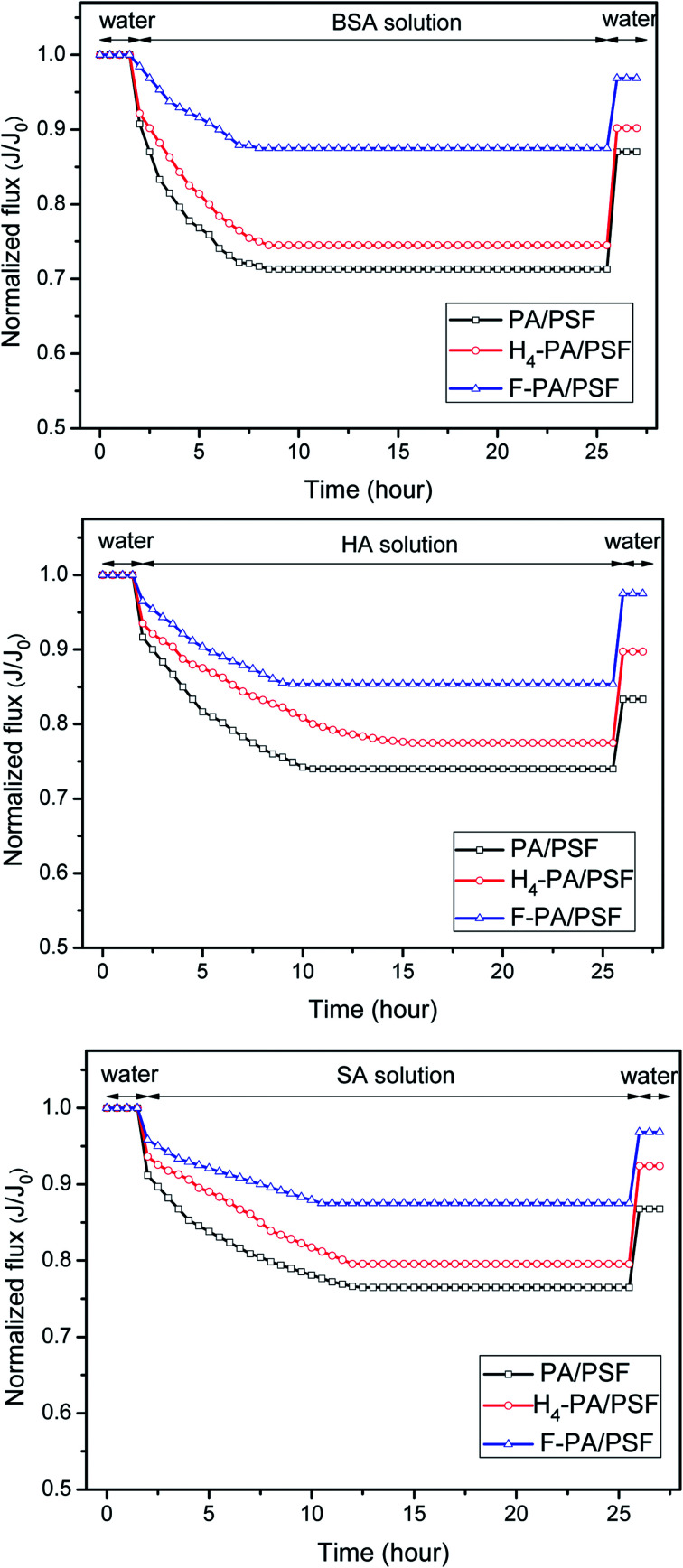
Time-dependent normalized fluxes for PA/PSF, H_4_-PA/PSF and F-PA/PES membranes during filtration of BSA solution; HA solution and SA solution, respectively, under the pressure of 1.0 MPa, temperature of 25.0 °C.

The time-dependent normalized flux (*J*_p_/*J*_w1_) profiles of the PA/PSF, H_4_-PA/PSF and F-PA/PSF membranes during filtration of aqueous solutions containing different model foulants are shown in Fig. 10. It is apparent that the fluxes of all membranes exhibited an obvious decline when a foulant solution was filtered. After some time, stable fluxes were obtained for all membranes. There are some interactions between membrane surface and foulants. On the one hand, the foulants deposit onto the membrane surface under the driving pressure and cause serious membrane fouling and flux decline. On the other hand, the foulants deposited on the membrane surface are removed from the membrane surface under the near surface shear stress. The flux tends to be stable when the adsorption and desorption of foulants reach to an equilibration under the joint effect of two kind of pressure. After this, a simple hydraulic cleaning was applied. The values (FRR, DR_t_) calculated from fluxes before and after fouling and cleaning were used to evaluate the antifouling properties of the membranes. FRR and DR_t_ values of PA/PSF, H_4_-PA/PSF and F-PA/PSF membranes for filtration of BSA, HA and SA aqueous solutions are given in Table 1. The FRR of the amphiphilic modified F-PA/PSF membrane is higher than that of the blank control PA/PSF membrane and the hydrophilic modified H_4_-PA/PSF membrane, and the DR_t_ is lower than that of the other two membranes during filtration of three different model foulant solutions. This manifests that the F-PA/PSF membrane has better antifouling properties. The good antifouling ability can be mainly ascribed to the amphiphilic nature of the F-PA/PSF membrane. On the one hand, the hydrophilic domains are beneficial to bind water molecules and form a compact hydration layer to alleviate the interaction between the membrane surface and potential foulants (fouling-resistant defense mechanism). On the other hand, the foulants can be readily detached under the hydrodynamic shear forces in the presence of hydrophobic segments with low surface energy barriers (fouling-release defense mechanism).

**Table 1:** Flux recovery ratio (FRR) and total flux decline ratio (DR_t_) values of the membranes in antifouling experiments of BSA, HA and SA aqueous solutions

Membrane	FRR (%)			DR_t_ (%)		
	BSA	HA	SA	BSA	HA	SA
PA/PSF	87.0	83.3	86.8	28.7	26.0	23.5
H_4_-PA/PSF	90.2	89.8	92.4	25.4	22.5	20.43
F-PA/PSF	96.9	97.5	96.8	12.5	14.6	12.5

**Fig. 11:**
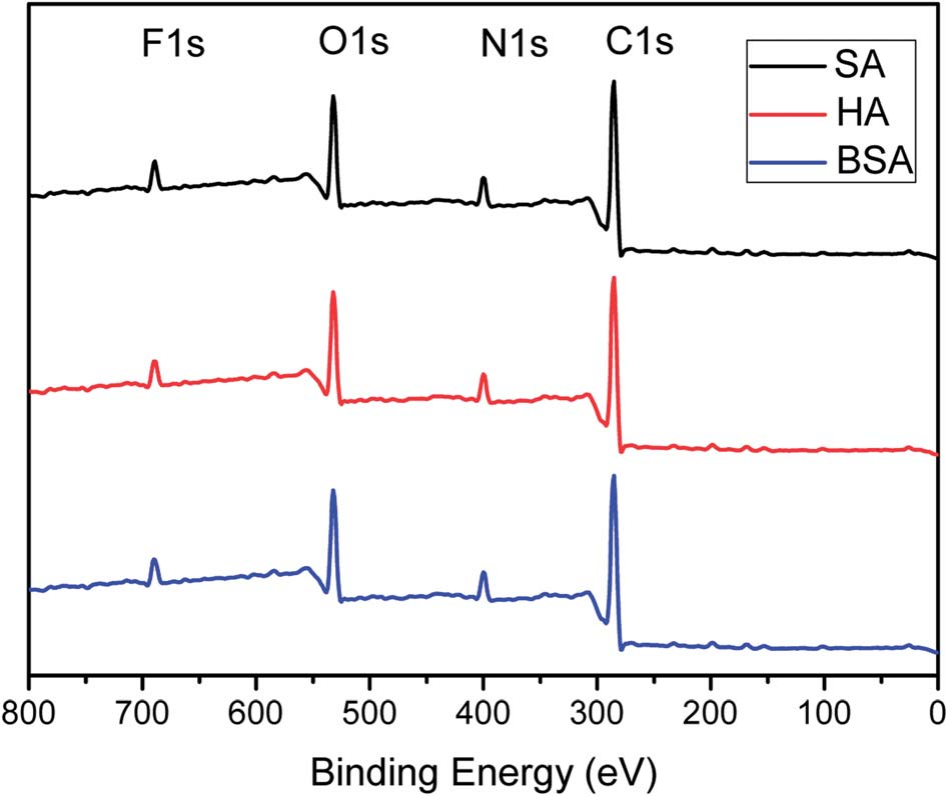
XPS scans of membranes of F-PA/PSF membrane after foulants (BSA, HA, SA) filtration and hydraulic cleaning.

To make sure the PFTS was not washed out during the high-pressure filtration testing or the hydraulic cleaning in the antifouling test. XPS characterizations of the filtrated and the fouled-cleaned F-PA/PSF membranes was further carried out to verify the existence of PFTS. The result was depicted in Fig. 11. Compared with the Fig. 3(a), the presence of F1s signal meant the stable existence of PFTS. So the amphiphilic modification of NF membrane was fairly stable under foulants filtration experiment and hydraulic cleaning.

## 4. Conclusions

In this study, a simple and effective method was employed to fabricate an amphiphilic NF membrane with hydrophilic domains and low surface energy domains through a two-step surface modification. The surface modification resulted in an unimportant decrease of the MWCO from 305 Da to 290 Da, while the Na_2_SO_4_ rejection increased slightly from 80.0% ± 0.8 (PA/PSF) and 82.3% ± 1.1 (H_4_-PA/PSF) to 82.9% ± 1.3, and maintaining a constant NaCl rejection of around 30%. Compared with the control PA/PSF membrane and the hydrophilic modified H_4_-PA/PSF membrane, the antifouling properties of the amphiphilic modified F-PA/PSF membrane were clearly enhanced. FRR values were 96.9%, 97.5%, and 96.8% for a BSA solution, HA solution and SA solution, respectively. As expected, the two-step surface modification did not alter the pore size of membranes. The membrane after amphiphilic modification was fairly stable, and PFTS on the surface were not washed out throughout the experiment. In addition, it should be noted that the introduction of hydrophobic PFTS in F-PA/PSF membrane promotes the water flux decline. Hence, this investigation can provide a method for fabrication of antifouling NF membranes under mild conditions.

## Conflicts of interest

There are no conflicts to declare.

## Supplementary Material
